# Flame-Retardant and Sound-Absorption Properties of Composites Based on Kapok Fiber

**DOI:** 10.3390/ma13122845

**Published:** 2020-06-25

**Authors:** Lihua Lyu, Yuanyuan Tian, Jing Lu, Xiaoqing Xiong, Jing Guo

**Affiliations:** School of Textile and Material Engineering, Dalian Polytechnic University, Dalian 116034, China; tian18940969545@163.com (Y.T.); lu18742057809@163.com (J.L.); xiongxq@dlpu.edu.cn (X.X.)

**Keywords:** kapok fiber, sound-absorption properties, flame-retardant properties, sound-absorbing mechanism

## Abstract

In order to improve the utilization rate of kapok fiber, flame-retardant and sound-absorption composites were prepared by the hot pressing method with kapok fiber as the reinforced material, polyε-caprolactone as the matrix material, and magnesium hydroxide as the flame retardant. Then, the effects of hot pressing temperature, hot pressing time, density of composites, mass fraction of kapok fiber, thickness of composites, and air layer thickness on the sound-absorption properties of composites were analyzed, with the average sound absorption coefficient as the index. Under the optimal process parameters, the maximum sound absorption coefficient reached 0.830, the average sound absorption coefficient was 0.520, and the sound-absorption band was wide. Thus, the composites belonged to high-efficiency sound-absorbing material. The flame-retardant effect of magnesium hydroxide on the composites was investigated, and the limiting oxygen index could reach 31.5%. Finally, multifunctional composites based on kapok fiber with flame retardant properties, and sound-absorption properties were obtained.

## 1. Introduction

As the world becomes more scientific and technological and the population grows, the distance between people is getting closer and closer, and the need to reduce and control noise has become more and more important. The need for quieter environments for consumers’ growing demands for personal quality of life and comfort has prompted designers and researchers to produce better noise control methods and materials and to adopt the principle of sustainable development to make people consider environmentally friendly materials [1]. Kapok fiber is a natural fiber, which has the advantages of softness, high hollow ratio, antibacterial, and mite removal, etc. It has received extensive attention and has good development prospect in the application of the textile field [2]. However, kapok fiber also has obvious disadvantages such as short length, poor strength, weak elasticity, and poor cohesion. Due to these shortcomings, kapok fiber cannot be spun alone, resulting in poor application in the field of clothing. At present, through processing, kapok fiber sound-absorbing materials can be developed.

Kapok fiber can be used as sound absorbing materials. Germany’s Dresden University of Technology [3] focused on the research of kapok fiber and wool fiber. Through experiments, the differences in heat insulation and sound insulation between the two were compared, and finally, it was concluded that kapok fiber material had better heat insulation and sound insulation performance than wool fiber material. Liu [4] developed a sound-absorbing nonwoven composite based on kapok fiber and hollow polyester fiber, and studied the sound-absorbing properties of a kapok fiber nonwoven composite in the low frequency region of 100–500 Hz by the impedance tube method. Xiang et al. [5] specifically explained the sound absorption properties of kapok fiber materials. The sound absorption properties of kapok fibers with different volume densities, lengths, thicknesses, and weights can be evaluated through impedance tubes. The experimental results showed that the hollow structure inside kapok fiber was the main reason for its good sound absorption properties. Second, the thickness, weight, and bulk density of kapok fiber also had certain effects on its sound-absorption properties, while the length had little effect on it. Compared with commercial glass wool and absorbent cotton fibers, kapok fibers with a smaller thickness could achieve the same sound-absorption effect as the other two. Makki [6] was used to combine kapok fiber with warp knitted fabric and double weave fabric in layers to study its sound absorption properties. The results showed that fiber material, material thickness, and material density were the main factors affecting the sound-absorption properties. Liu et al. [7] studied the sound-absorption properties of kapok fiber composites at low frequency. The results showed that the sound-absorption properties at low frequency could be improved by increasing the thickness of the rear air layer. At present, it is very necessary to improve the sound absorption properties of kapok fiber materials at low frequencies.

Although kapok fiber can be used as sound-absorbing material, it has a serious disadvantage in that it is flammable. Chung et al. [8] used gamma rays to remove combustible compounds in kapok fibers and cracked functional groups in lignin polymers to realize the flame retardant properties of kapok fibers. Wu et al. [9] encapsulated phosphorus flame retardant into kapok fiber to form capsule-shaped tubules. The results showed that through thermogravimetric analysis and cone calorimetry analysis, the microtubules obviously reduce heat release, oxygen consumption, and smoke exhaust during combustion because the microtubules form a protective layer on the combustion surface to achieve the purpose of a flame retardant. Kwon et al. [10] invented a flame retardant that can be used to treat kapok composites, and showed that it can be used in automobiles, clothing, bedding, etc.

Polycaprolactone (PCL) is biodegradable, so it is widely used in many fields of application. PCL is usually used in the fields of biomedical applications [11], conducting fibers [12], and compostable plastics [13], but mostly used in composites [14]. Scaffaro et al. [15] prepared biopolymer porous devices with gradient properties-multiphase porous laminates using PCL and polylactic acid as binders, and characterized all materials from the perspectives of morphology and mechanics. Chen et al. [16] studied completely bioabsorbable composites with phosphate-based glass fiber (PGF) reinforced polycaprolactone (PCL) composites. The reinforcing efficiency of laminate stacking and in situ polymerization process was compared. The results showed that the in situ polymerization process had better reinforcing efficiency for the study of degradation and mechanical properties. Dhakal et al. [17] prepared biological composites with PCL as the matrix material and hemp fiber as the reinforcement material. The effect of fiber aspect ratio on the water absorption and mechanical properties of the composite was studied. The results showed that the moisture absorption percentage increased with the increase of aspect ratio. When the aspect ratio was 26, the bending strength and bending modulus of the hemp fiber/PCL biological composite were higher than those of pure PCL. Liu et al. [18] used PCL and poplar seed fiber to prepare sound-absorption composites, and the results showed that the sound-absorption coefficient of composites higher than 0.7 PCL could be used as the matrix material of the sound-absorbing composites. These references [14,15,16,17,18] showed that polycaprolactone had good compostable character and compounding property, so it could be used as a matrix of the composites. PCL’s low melting point and easy combustion hinder its application in many fields. Chen [19] studied an intumescent flame retardant with better proportions of phosphorus, nitrogen, and carbon, and prepared a flame retardant PCL containing phosphorus biomass material. The results showed that the addition of intumescent flame retardant can improve the flame retardant performance of PCL. PCL also has biodegradability and can be used to prepare environmentally friendly materials.

The main purpose of this paper was to study the flame-retardant and sound-absorption properties of the composites with kapok fiber as the reinforced material, PCL as the matrix material, and magnesium hydroxide as the flame retardant through range analysis and single factor experiment. Finally, a multifunctional composite based on kapok fiber with flame retardant properties and sound absorption properties was obtained.

## 2. Experiment

### 2.1. Materials and Equipment

Kapok fibers with a length of 8–34 mm, a density of 290 kg/m^3^ (CUHK Textile Co. Ltd., Guangzhou, China), 800 mesh polyε-caprolactone (PCL), melting point of 59–64 °C, molecular weight of 50,000 (American Suwei Co. Ltd., Dongwan, China); magnesium hydroxide of 2–5 µm (Jinan Taixing Fine Chemical Co. Ltd., Jinan, China); antimony trioxide of 1.1 µm (Shandong Yousuo Chemical Technology Co. Ltd., Linyi, China); 500 mesh ammonium polyphosphate (Changzhou Phosphorus Chemical Co. Ltd., Changzhou, China); and zinc borate with a molecular weight of 400 (Shandong Yousuo Chemical Technology Co., Ltd., Linyi, China) were used as the raw materials.

A scanning electron microscope, (JEOL JSM-6460LV, Japan Electronics Co. Ltd., Beijing, China) was used to observe the surface structure of the materials. An electronic balance, FA2004B, (Shanghai Precision Experimental Instrument Company, Shanghai, China); pressure forming machine, QLB-50D/QMN (Jiangsu Wuxi Zhongkai Rubber and Plastic Machinery Co. Ltd., Wuxi, China); Standing Wave Tube/Impedance Tube Sound Absorption and Sound Insulation Test Systems SW422/SW477, (Beijing Shengwang Electronic Technology Co. Ltd., Beijing, China); and LFY606b digital oxygen index tester (Shandong Textile Research Institute) were used for testing.

### 2.2. Preparation Technology of Composites

#### 2.2.1. Preparation of Sound-Absorbing Composites

Kapok fiber and PCL were mixed and hot pressed by the QLB-50D/QMN pressure pressing machine. The samples were first prepared at the hot pressing time of 900 s, density of 188 kg/m^3^, mass fraction of kapok fiber of 50%, and thickness of 10 mm. The hot pressing process parameters were as follows: hot pressing temperatures were 373.15 K, 403.15 K, 433.15 K, and 463.15 K; hot pressing pressure was 10^7^ Pa; hot pressing time was 300 s and 900 s. Samples with different structural parameters were prepared by single factor experimental analysis. When different densities of composites were prepared, the mass fraction of kapok was 50% and the thickness of the samples was 10 mm. The designed densities of composites were 156 kg/m^3^, 172 kg/m^3^, 188 kg/m^3^, and 205 kg/m^3^. When different mass fractions of kapok were prepared, the thickness of the sample was 10 mm. Samples with 30%, 40%, 50%, and 60% of kapok fiber mass fraction were prepared. When the composites were prepared with different thicknesses, the thicknesses of the samples were 10 mm, 15 mm, 20 mm, and 25 mm. When we studied the influence of the thickness of air layer on the sound-absorption properties, (the thickness of the rear air layer is to separate the kapok fiber sound-absorption material from the rigid wall by a specific distance), the air layer with different thicknesses (the thicknesses were 0 mm, 5 mm, 10 mm, 15 mm, and 20 mm, respectively) was arranged behind the sample. The sizes of the composites were the same as that of the impedance tube, with diameters of 100 mm and 30 mm. The melting point of PCL is 59–64 °C. Since the hot pressing method was primitive, the mixing of PCL and fibers was not uniform ideally, so centrifugal spinning and pressurized gyration could be used instead of this method to realize the uniform mixing of PCL and the fibers, and the manufacturing of the fiber mat was less than 1 s, which could improve the yield of nonwoven fiber mats [20].

#### 2.2.2. Preparation of Flame Retardant Treatment for Kapok Fiber

Weigh the quantity of kapok and PCL according to the experimental scheme. Add flame retardant and mix the raw materials. As shown in Table 1, experimental formula for adding the flame retardant. Press the mixed raw materials onto a fixed template, spray a mold release agent on the upper and lower interfaces of the mold, and hot press the filled materials according to the experimental plan; then place the formwork between the two iron pressing plates and stand, cool in an air vent with air circulation, and demold after 300–1200 s. Next, take out the sample when cooling to a certain temperature. The splines made from the prepared template were cut by the cutting prototype for testing.

### 2.3. Testing of Composites

#### 2.3.1. Testing of Sound-Absorption Properties

**Figure 1:**
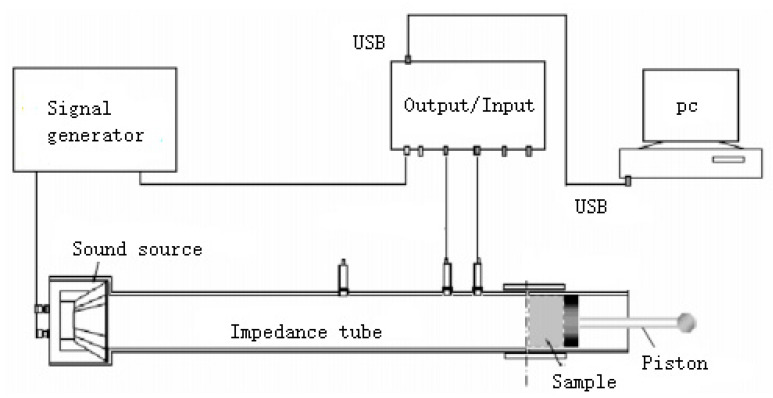
Schematic diagram of the sound-absorption test.

The SW422/SW477 impedance tube sound absorption test system is shown in Figure 1. The impedance tube is a metal cylindrical tube with no other friction on the inner wall, and an inner diameter of 100 mm (for low and medium frequencies) or 30 mm (for high frequencies), respectively. Random sound waves were generated by speakers at the end of the impedance tube, and then the waves were transmitted to the sample surface at the other end. The reflected signal was then received by the sensor. The large tube can measure the sound-absorption coefficient in the frequency range of 80–2500 Hz, while the small tube can generate the sound-absorption coefficient in the frequency range of 1600–6300 Hz. The sound-absorption coefficient in the full frequency range (80–6300 Hz) is a combination of the values measured in the two tubes. During the experimental measurement, the samples were all supported by rigid walls.

The test standards for sound-absorption properties are IS010534-2: 1998 and GB/T1869.2-2002, and the test method is the transfer function method. The test environment was set at a temperature of 25 °C, relative humidity of 65%, atmospheric pressure of 101.325 Pa, and sound speed of 346.11 m/s. The absorption property can be characterized by that average absorption coefficient average sound-absorption coefficient: the arithmetic average of six sound-absorption coefficients at frequencies of 125 Hz, 250 Hz, 500 Hz, 1000 Hz, 2000 Hz, and 4000 Hz.

#### 2.3.2. Testing of Flame-Retardant Properties

**Figure 2:**
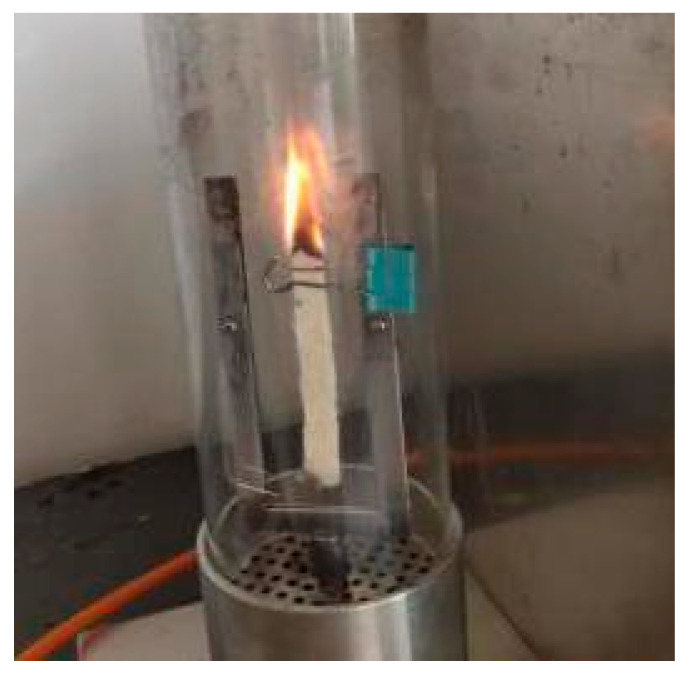
Testing of flame-retardant properties of the composites.

The experiments of the flame-retardant properties were carried out according to the GB/T8924-2005 “Test Method for Combustion Performance of Fiber Reinforced Plastics Oxygen Index Method” standard. The sample size was 12 mm × 10 mm × 4 mm. Limiting oxygen index (LOI) is used to characterize the flame retardant properties of composites where the higher the limit oxygen index, the better the flame-retardant property. When the measured oxygen index was 22~25%, the test sample was flammable. The oxygen index was 26~30%, and the detected sample was flame retardant; when the oxygen index was above 30%, the flame retardant properties were better. The testing of the flame-retardant properties of the composites is shown in Figure 2.

## 3. Results and Discussion

### 3.1. Sound-Absorption Properties

#### 3.1.1. Influence of Hot Pressing Temperature on Sound-Absorption Properties

**Figure 3:**
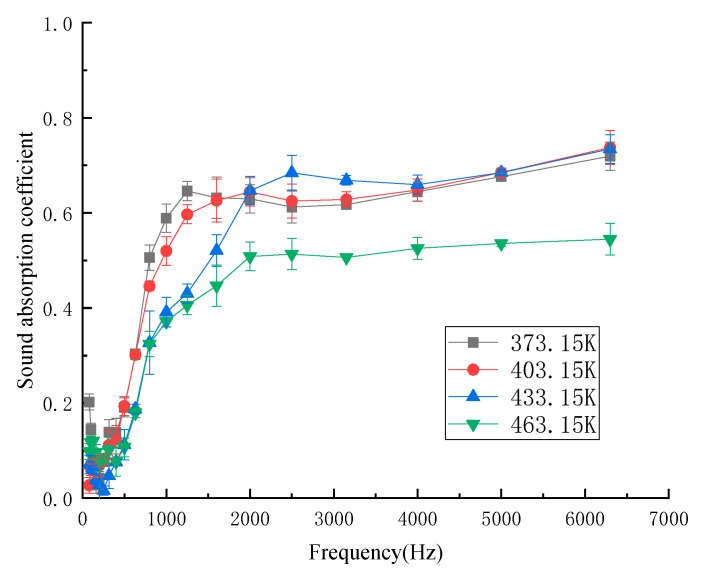
Sound-absorption coefficient at different hot pressing temperatures.

The influence of hot pressing temperature on the sound-absorption coefficient was determined when the hot pressing time was 900 s, the density was 188 kg/m^3^, the mass fraction of kapok fiber was 50%, and the thickness was 10 mm. Figure 3 shows the influence of different hot pressing temperatures on the sound-absorption coefficient. The sound-absorption frequency range was 80–6300 Hz, covering the low frequency range (200–2000 Hz), intermediate frequency range (2000–4000 Hz), and medium high frequency range (4000–6300 Hz). From Figure 3, the low frequency and sound-absorption properties of composites were not good at the four temperatures. With the increase in temperature in the middle and low frequency region, the sound-absorption coefficient gradually decreased. In the intermediate frequency region, the sound-absorption coefficient started to exceed at 373.15 K, 403.15 K, and 433.15 K. However, the sound absorption coefficient of 463.15 K was still not very good. The sound-absorption coefficient fluctuated slightly in the medium and high frequency regions, but the sound-absorption properties were very good at all three temperatures. The average sound-absorption coefficient of kapok fiber composites at the four hot pressing temperatures was 0.352 (373.15 K), 0.358 (403.15 K), 0.318 (433.15 K), and 0.288 (463.15 K). The average sound-absorption coefficient of the composites at 403.15 K was higher than those with other hot pressing temperatures. At the temperature of 403.15 K, the adhesion between the matrix material and kapok fiber was good, and its pores were more uniform [21]. Therefore, we considered the forming and sound-absorption coefficient of the composites at the hot pressing temperature of 403.15 K.

#### 3.1.2. Influence of Hot Pressing Time on Sound-Absorption Properties

**Figure 4:**
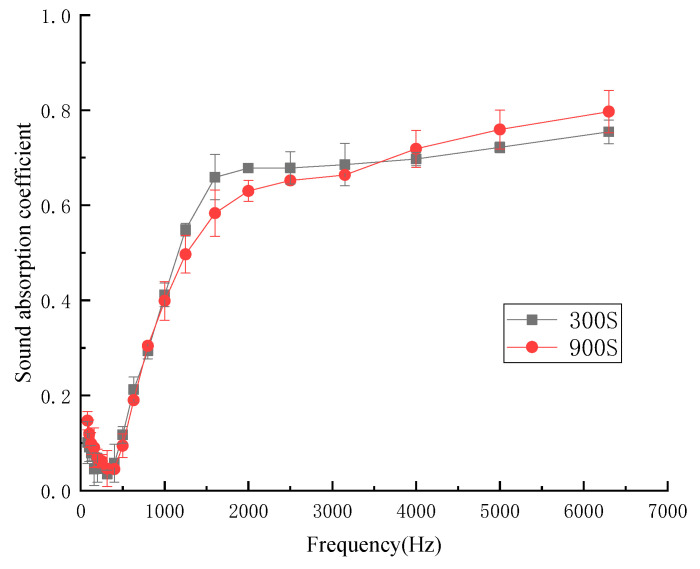
Sound-absorption coefficient at different hot pressing times.

The influence of hot pressing time on sound-absorption coefficient was determined by setting the hot pressing time as 300 s and 900 s. Figure 4 shows the influence of different hot pressing times on the sound-absorption coefficient.

Under the two times, there was almost no difference in the sound-absorption coefficients of kapok fiber composites at low frequency, and medium and low frequency. There were gradual differences in the intermediate frequency, medium high frequency, and high frequency regions. When the hot pressing time was too short, the kapok fiber composites were difficult to form and their sizes were unstable, so the following experiments could not be further expanded. When the hot pressing time was appropriate, the hot pressing effect on the composites was that the materials were pressed more evenly, and more air voids inside the materials were distributed evenly. The average sound-absorption coefficient of the fiber composites with different hot pressing times was 0.330 (300 s) and 0.330 (900 s). Considering the molding stability of composites, the hot pressing time of 900 s was the best process parameter.

#### 3.1.3. Influence of Density of Composites on Sound-Absorption Properties

**Figure 5:**
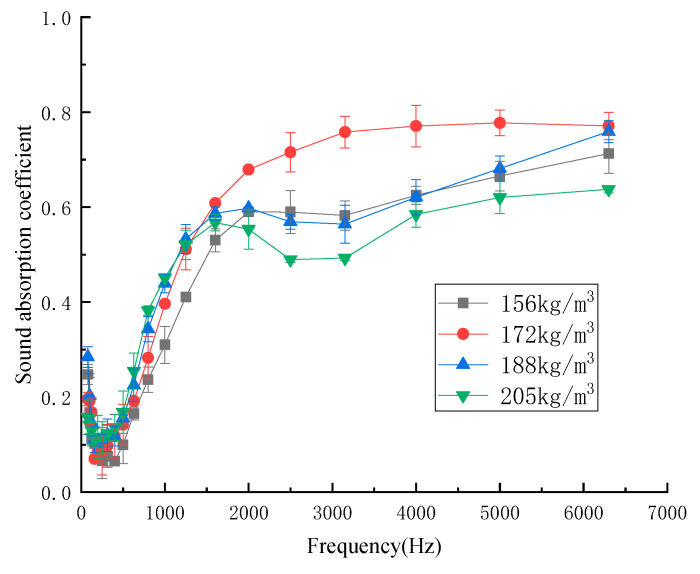
Sound absorption coefficient with different densities.

The sound-absorption properties depend on the density of the material [22]. To study the relationship between the sound-absorption properties and the density of the kapok fiber composites, the design densities of the composites were 156 kg/m^3^, 172 kg/m^3^, 188 kg/m^3^, and 205 kg/m^3^. Figure 5 shows the influence of the density of composites on the sound-absorption coefficient.

As shown in Figure 5, the sound-absorption coefficient increased first and then decreased with the increase in density. The average sound-absorption coefficient of kapok fiber composites with different densities was 0.303 (156 kg/m^3^), 0.365 (172 kg/m^3^), 0.347 (188 kg/m^3^), and 0.323 (205 kg/m^3^). When the density was 172 kg/m^3^, the average sound absorption coefficient reached 0.388, and the sound absorption band range was the largest. When the density was 172 kg/m^3^, the number of micropores in the composites was relatively greater. When the density was less than 172 kg/m^3^, with the increase in the density of the composites, the number of micropores in the composites with the same thickness increased, thus improving the sound-absorption properties of the composites. However, with the increased density of the composites, the pore size and number of micropores in the composites decreased, which reduced the friction and vibration between the air and fibers in the composites, thus reducing the consumption of sound energy and the sound-absorption properties [18]. According to the above analysis in Figure 5, the density of 172 kg/m^3^ was the best process parameter.

#### 3.1.4. Influence of Mass Fraction of Kapok Fiber on Sound-Absorption Properties

**Figure 6:**
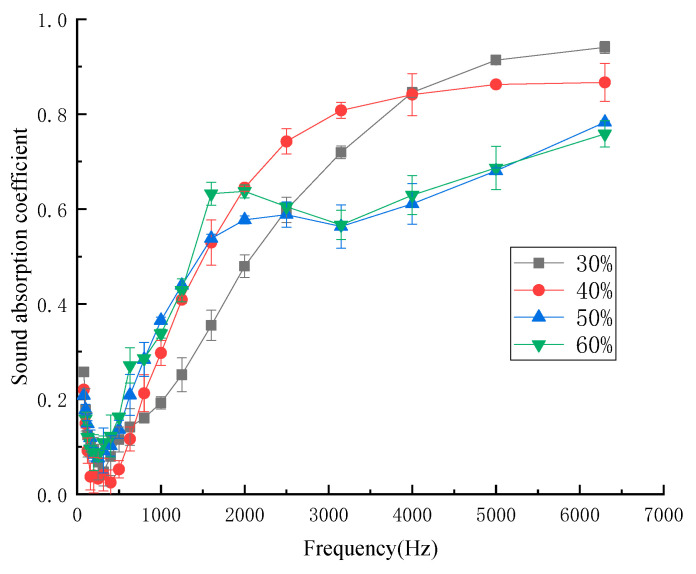
Sound-absorption coefficient with different mass fractions of kapok fiber.

In order to investigate the influence of the mass fraction of kapok fiber on sound-absorption performance, the samples had the mass fraction of kapok fiber of 30%, 40%, 50%, and 60%. Figure 6 shows the influence of the different mass fractions of kapok fiber on the sound-absorption coefficient.

**Figure 7:**
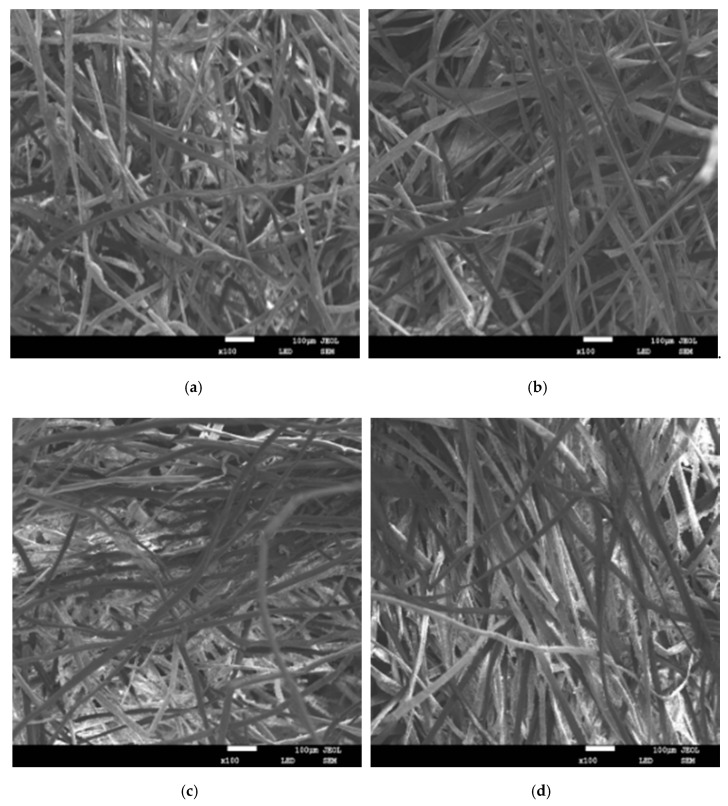
Scanning electron microscope (SEM) images of composites with different mass fractions of kapok fiber. (**a**) Mass fraction of kapok fiber of 30% (**b**) Mass fraction of kapok fiber of 40%. (**c**) Mass fraction of kapok fiber of 50% (**d**) Mass fraction of kapok fiber of 60%.

As shown in Figure 6, in the low frequency and medium low frequency regions, with the increase in the mass fraction of kapok fiber, the sound-absorption coefficient increased within a certain range because the sound-absorption performance of the composite depends on the content of the fiber. When the content of the fiber is higher, the surface area of the fiber is larger, so the greater the interaction between the sound wave and fiber [23]. In the medium and high frequency regions, the sound-absorption effect worsened with large fiber mass fraction. When the mass fraction of kapok fiber was 30%, the maximum sound-absorption coefficient of the sample was 0.920. However, the sound-absorption properties of the sample were not good at medium and low frequencies, and only good at high frequencies. The average sound-absorption coefficient with different mass fractions of kapok fiber was 0.304 (30%), 0,323 (40%), 0.319 (50%), 0.314 (60%). This is because compared with other composites with different mass fractions of kapok fiber, when the mass fraction of kapok fiber was 40%, the number of micropore structures formed was greater. As can be seen in Figure 7, when the mass fraction of kapok fiber was 30%, the micropore structure formed by the composite material was larger, which made the sound energy penetrate more, and the sound-absorption properties were poor. When the mass fraction of kapok fibers was 50% and 60%, more kapok fibers were extruded together and the number of micropores formed was reduced, which reduced the friction and vibration between the sound energy and kapok fibers and reduced the sound-absorption properties. These results correspond with the experimental data of Tian et al., who reported that when the mass fraction of kapok fiber was 40%, the average sound-absorption coefficient was 0.398 [19]. According to the above analysis from Figure 6 and Figure 7, the mass fraction of kapok fiber was 40%.

#### 3.1.5. Influence of Thicknesses on Sound-Absorption Properties

**Figure 8:**
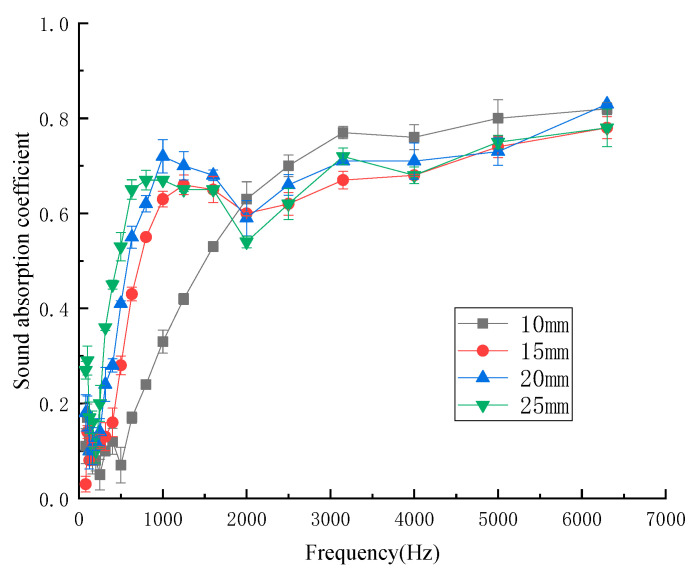
Sound-absorption coefficient with different thicknesses.

The thickness of the material plays an important role in the sound-absorption coefficient [24]. To study the relationship between the sound-absorption properties and the thickness of the kapok fiber composites, the thicknesses of the samples were 10 mm, 15 mm, 20 mm, and 25 mm, respectively. Figure 8 shows the influence of different thicknesses of kapok fiber composites on the sound-absorption coefficient.

As shown in Figure 8, when the thickness was 15 mm, the peak frequency of the sound-absorption coefficient was 1600 Hz, and when the thickness was increased to 25 mm, the peak frequency of the sound-absorption coefficient was 800 Hz. Thus, with the increase in thickness, the peak value of the sound-absorption coefficient moved to the lower frequency band. With the increase in material thickness, the air permeability decreased, the flow resistance increased, and the sound-absorption effect was enhanced. The lower the thickness, the shorter the time and distance through the channel, so the sound wave cannot be reflected and refracted many times, and the sound-absorption coefficient of the material decreased. Therefore, thickness is generally considered as an important factor to control sound-absorption performance. However, with that thickness of kapok fiber composite to a certain extent, the sound-absorption coefficient no longer changed. The average sound-absorption coefficient of different thicknesses of kapok fiber composites was 0.327 (10 mm), 0.398 (15 mm), 0.445 (20 mm), and 0.465 (25 mm). The average sound absorption coefficient of kapok fiber composites increased with the increase in thickness, and the increase trend was obvious. As a result, the energy consumed by acoustic energy entering the composite and friction with fibers increased, and there was also a long viscous heat conduction dissipation process between the air inside the composites and the composites themselves [25]. Considering the material cost, the thickness of the composites was 20 mm.

#### 3.1.6. Influence of Air Layer Thickness on Sound-Absorption Properties

**Figure 9:**
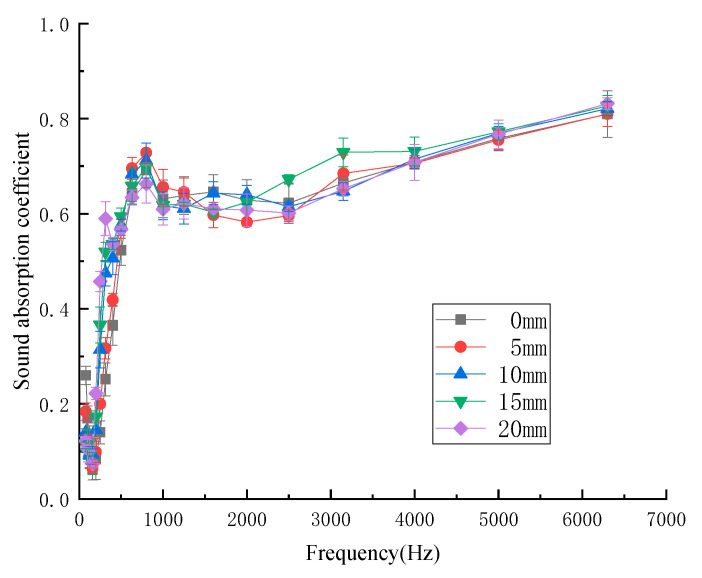
Sound-absorption coefficient with different air layer thicknesses.

The thickness of the air layer behind the composite materials was a certain distance between the kapok fiber composites and the rigid wall. In order to investigate the influence of different air layer thickness on sound-absorption properties, air layers with different thicknesses (thickness 0, 5, 10, 15, 20 mm, respectively) were set behind the samples to test the sound absorption coefficient. Figure 9 shows the influence of air layer with different thicknesses of kapok fiber composites on the sound absorption coefficient. As shown in Figure 9, the influence trend of the air layer thickness on the sound-absorption properties was roughly the same as that of increasing the thickness of the composites, that is, with the increase in the thickness of the air layer of the sample, the peak value of the sound-absorption coefficient moved to low frequency, and the sound-absorption coefficient decreased at high frequency. Therefore, increasing the thickness of the air layer after the material was equivalent to increasing the thickness of the composites. The average sound-absorption coefficient of the air layer with different thicknesses of kapok fiber composites was 0.440 (0 mm), 0.460 (5 mm), 0.483 (10 mm), 0.498 (15 mm), and 0.520 (20 mm). When the thickness of the air layer was 20 mm, the average sound-absorption coefficient could reach 0.520.

#### 3.1.7. Sound-Absorption Properties of Composites under Optimal Process Parameters

**Figure 10:**
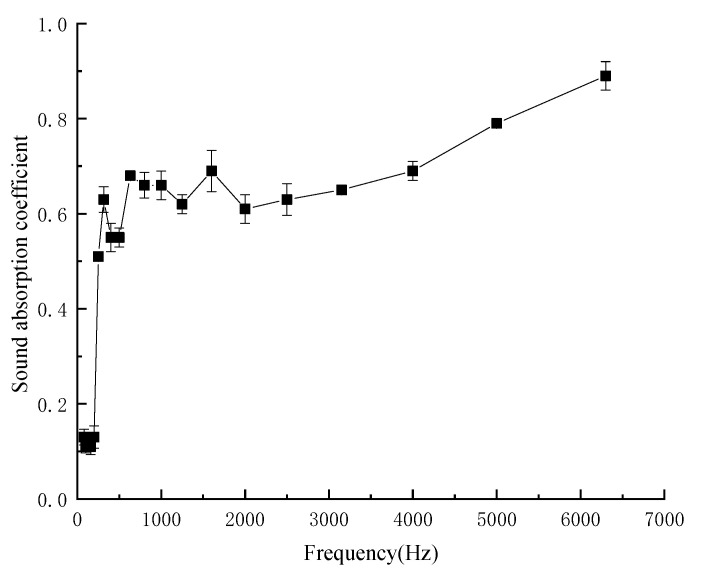
Sound-absorption coefficient of composites under optimal process parameters.

The optimum process parameters for preparing kapok fiber composites were as follows: hot pressing temperature of 403.15 K, hot pressing time of 900 s, density of composites of 172 kg/m^3^, mass fraction of kapok fiber of 40%, thickness of 20 mm, and air layer thickness of 20 mm. As shown in Figure 10, sound-absorption coefficient of composites under optimal process parameters. Under the optimal process parameters, the maximum sound absorption coefficient reached 0.830, the average sound absorption coefficient was 0.520 and the sound absorption band was wide. Therefore, the composites belonged to the high-efficiency sound-absorbing material. The environmentally-friendly sound-absorption materials also had antibacterial properties. Su et al. [26] analyzed the chemical composition of kapok fibers and believed that the reason why kapok was antibacterial was that it contained flavonoids and triterpenes. Another reason may be due to the hollow structure of the kapok fiber having many micropores on its surface, which makes the kapok fiber rich in oxygen and had good moisture absorption and air permeability, which means that anaerobic bacteria could not survive on kapok fiber. At the same time, under humid conditions, the reproductive metabolism and physiological activities of mold microorganisms growing on kapok fiber will also be inhibited, effectively inhibiting oxidative phosphorylation of microorganisms, affecting mitosis, and hindering microbial respiration. Therefore, kapok fiber shows an antibacterial effect [27].

### 3.2. Flame Retardant Properties

Magnesium hydroxide is decomposed into magnesium oxide and water at about 613.15 K, which will absorb a large amount of heat in the reaction process. Research has shown that 1400 J of heat needs to be provided when 1 g magnesium hydroxide is decomposed, and the water generated by the reaction will also absorb some heat, thus reducing the temperature of the combustible substrate and slowing down the combustion speed [28]. The water vapor generated by decomposition can dilute the concentration of combustible gas (oxygen) on one hand, and form protective gas around the substrate on the other hand, which plays a flame retardant role between the gas phase region and the solid phase region. The magnesium oxide generated by decomposition will be pasted on the surface of the substrate to form an adiabatic shielding layer, and the spread rate of the thermal decomposition gas from the solid phase to the combustion zone will be greatly reduced, thus inhibiting combustion. Synergistic agents such as zinc borate can be used as flame retardants and smoke suppressants, which can improve the flame retardant properties of composites [29]. The formulation of the flame retardant was in accordance with Section 2.2.2, and the experimental results are shown in Table 2. The addition of magnesium hydroxide significantly reduced the elongation at break of the composites. Lan tested the elongation at break and tensile strength of pure EVA (Ethylene-vinyl acetate) and magnesium hydroxide/EVA composites, the elongation at break of pure EVA was 810.45%, when adding 30 wt% of magnesium hydroxide, the elongation at break of magnesium hydroxide/EVA composites was 619.69%, which was 23.5% lower than that of pure EVA. With the addition of magnesium hydroxide from 30 wt% to 60 wt%, the elongation at break of composites decreased to 183.91%, and the tensile strength also decreased from 27.5 MPa to 14.55 MPa, which was 47.0% lower than that of pure EVA. This result showed that the addition of magnesium hydroxide over 30% had great influence on the elongation at break and the tensile strength of the composites [30]. Therefore, the amount of magnesium hydroxide added should not exceed 30%. Flame retardant treatment was carried out on the composites with the optimum process parameters in Section 3.1.7.

As shown in Table 2, when only the magnesium hydroxide flame retardant was added, the LOI value increased continuously with the increase of the added amount, showing a linear growth trend. Therefore, the larger proportion of magnesium hydroxide added, the better the flame retardant properties of the composites. However, in the magnesium hydroxide compound system, zinc borate and antimony trioxide showed a good synergistic flame retardant effect, while ammonium polyphosphate had no obvious synergistic flame retardant effect. This is because magnesium hydroxide and the first two cooperate to form a dense carbon layer, which can block external heat sources and also prevent combustible gas (CO) generated inside the material from contacting external oxygen when the temperature rises and continues to be heated, thus forming a flame retardant. Therefore, the formula of 30% magnesium hydroxide and 5% antimony trioxide was finally selected to prepare the kapok fiber flame-retardant and sound-absorption composites.

## 4. Conclusions

The optimum pressing parameters for preparing the flame retardant and sound-absorbing composites of kapok fiber are as follows: hot pressing temperature at 403.15 K, hot pressing time of 900 s, density of the nonwoven material of 172 kg/m^3^, mass fraction of kapok of 40%, thickness of 20 mm, and the thickness of air layer of 20 mm. Under the optimal process parameters, the maximum sound-absorption coefficient reached 0.830, the average sound-absorption coefficient was 0.520, and the sound-absorption band was wide. Therefore, the composites belonged in the category of a high-efficiency sound-absorbing material.

Flame-retardant and sound-absorption composites of kapok fiber were prepared. We added 30% magnesium hydroxide and 5% antimony trioxide to the formula to synergistically act as a flame retardant, and its limiting oxygen index was measured to reach 31.5%.

Environmentally-friendly composites were developed in this research. The composites’ sound-absorption and flame retardant properties were tested and the results showed that PCL/kapok fiber composites can be applied to building materials.

## Figures and Tables

**Table 1:** Experimental formula for adding the flame retardant.

Samples	Magnesium Hydroxide (%)	Zinc Borate (%)	Ammonium Polyphosphate (%)	Antimony Trioxide (%)
1	20	0	0	0
2	25	0	0	0
3	30	0	0	0
4	35	0	0	0
5	40	0	0	0
6	45	0	0	0
7	50	0	0	0
8	30	5	0	0
9	30	0	5	0
10	30	0	0	5

**Table 2:** Flame retardant properties of kapok fiber composites.

Samples	Magnesium Hydroxide (%)	Zinc Borate (%)	Ammonium Polyphosphate (%)	Antimony Trioxide (%)	LOI (%)
1	20	0	0	0	26.0
2	25	0	0	0	26.5
3	30	0	0	0	27.0
4	35	0	0	0	27.5
5	40	0	0	0	28.5
6	45	0	0	0	28.5
7	50	0	0	0	29.5
8	30	5	0	0	30.0
9	30	0	5	0	28.0
10	30	0	0	5	31.5
